# A Genome Scan for Selection Signatures in Pigs

**DOI:** 10.1371/journal.pone.0116850

**Published:** 2015-03-10

**Authors:** Yunlong Ma, Julong Wei, Qin Zhang, Lei Chen, Jinyong Wang, Jianfeng Liu, Xiangdong Ding

**Affiliations:** 1 Key Laboratory of Animal Genetics, Breeding and Reproduction, Ministry of Agriculture, National Engineering Laboratory for Animal Breeding, College Animal Science and Technology, China Agricultural University, Beijing, P.R. China; 2 Chongqing Academy of Animal Science, Chongqing, P.R. China

## Abstract

Identifying signatures of selection can provide a straightforward insight into the mechanism of artificial selection and further uncover the causal genes related to the phenotypic variation. Based on Illumina Porcine60KSNP chip data, four complementary methods, Long-Range Haplotype (LRH), Tajima’s D, Cross Population Extend Haplotype Homozygosity Test (XPEHH) and F_ST_, were implemented in this study to detect the selection signatures in the whole genome of one typical Chinese indigenous breed, Rongchang, one Chinese cultivated breed, Songliao, and two western breeds, Landrace and Yorkshire. False Discovery Rate (FDR) was implemented to control the false positive rates. In our study, a total of 159, 127, 179 and 159 candidate selection regions with average length of 0.80 Mb, 0.73 Mb, 0.78 Mb and 0.73 Mb were identified in Landrace, Rongchang, Songliao and Yorkshire, respectively, that span approximately 128.00 Mb, 92.38 Mb, 130.30 Mb and 115.40 Mb and account for approximately 3.74–5.33% of genome across all autosomes. The selection regions of 11.52 Mb shared by Landrace and Yorkshire were the longest when chosen pairs from the pool of the four breeds were examined. The overlaps between Yorkshire and Songliao, approximately 9.20 Mb, were greater than those of Yorkshire and Rongchang. Meanwhile, the overlaps between Landrace and Songliao were greater than those of Landrace and Rongchang but less than those of Songliao and Ronchang. Bioinformatics analysis showed that the genes/QTLs relevant to fertility, coat color, and ear morphology were found in candidate selection regions. Some genes, such as LEMD3, MC1R, KIT, TRHR etc. that were reported under selection, were confirmed in our study, and this analysis also demonstrated the diversity of breeds.

## Introduction

Looking back on the evolutionary history of the pig, the domestication process began almost simultaneously in separate areas of the Asia and Europe [1,2]. Since then, the pig has experienced evolution over a very long time under natural and artificial selection. Domestic pig phenotypes, including both production-relevant traits and behavior, have been largely transformed compared to its wild counterpart, and the phenotypic variation in the breeds also becomes more distinctive [3].

To better understand the underlying genetic mechanism for phenotypic distinction caused by selection in pig, the hunt for genomic evidence of selection has been performed in various pig breeds using whole genome genotype data or pool sequencing data [4–9]. The studies associated with growth traits, reproduction traits and even coat color, to which breeders had attached great importance, have shown that selection facilitates the homozygosity of beneficially allelic genes [4,5]. Several genes with major effects on growth, reproduction trait and coat color have already been identified under selection, like insulin-like growth factor 2 (IGF2), relevant to muscle growth [10], parathyroid hormone-like hormone (PTHLH), associated with litter size and the number of teats in European pig [5,11] and v-kit Hardy-Zuckerman 4 feline sarcoma viral oncogene homolog (KIT) and melanocortin 1 receptor (MC1R), related with a series of pig breed color types [4].

Theoretically, a novel beneficial variant that has been under selection pressure usually shows long-range linkage disequilibrium (LD) and a high population frequency over a long period of time [12]. Thus, selection signatures could be detected through the decay of linkage disequilibrium and the variation of allele frequency. These methods for detecting selection signatures can be grouped into three categories according to the information used: population differentiation, site-frequency spectrum and linkage disequilibrium [13,14]. Corresponding to these groups, the F_ST_, the Tajima’s D test, the Cross Population Extend Haplotype Homozygosity Test (XPEHH) and the long range haplotype (LRH) are the representative methods widely used in identifying selection signatures. Among them, F_ST_ was initially used to assess population differentiation according to the DNA polymorphism in populations, which was attributed to geographically variable selection [15–17]. Tajima’s D is the most famous method for detecting selection signatures based on segregating sites frequency, and it is sensitive to purifying selection and balancing selection [18]. LRH was developed to measure the degree of LD in one region with long range haplotypes with an adjustment to local variation in recombination rates, which is sensitive to the regions with a rapidly increased frequency of the derived allele at selected sites but may not detect selection at the fixed regions because of the elimination of variation at those sites [19]. Although both LRH and XPEHH are typical haplotype methods, XPEHH assumes that the occurrence of selection can be traced by measuring LD or observing overrepresented haplotypes in the observed population, making it possible to detect entirely or approximately fixed sites [19]. Recently, Rubin et al. [20] proposed a method ZH_P_ utilizing the pooled heterozygosity in small window to detect selection signatures, mainly dealing with pooling chip/sequencing data.

Although some studies have been carried out to detect selection signatures in the pig, the findings have not been totally concordant due to the limitations of sample size and statistical methodology. In addition, the selection patterns in pig breeds are different as their different evolution histories. Therefore, it is necessary to explore selection signatures in more pig breeds, which will be helpful to better understand the genetic variation in different pig breeds and identify common variants in traits of interest. In this study, we detected the selection signatures at the whole genome level in one Chinese indigenous breed, Rongchang, one Chinese cultivated breed, Songliao, and two western breeds, Landrace and Yorkshire, using Illumina porcineSNP60K BeadChip. Four methods, LRH, Tajima’s D, XPEHH and F_ST_ were implemented to identify selection signatures. Bioinformatics analysis was also performed to explain the biological function of the selection signatures.

## Materials and Methods

### Populations

A total of 338 individuals from four pig breeds were collected for the experimental population in our study, including 72 Rongchang (Chinese indigenous breed), 86 Songliao (Chinese cultivated breed), 97 Yorkshire and 83 Landrace pigs. As a typical representative of indigenous breed, Rongchang was breed in the southwest of China and has a special coat color in comparison with other Chinese indigenous breeds. In the past decades, Songliao was cultivated through hybridization of the Duroc, Landrace and Chinese Minzhu, which originated in Northeast of China. Landrace and Yorkshire are superior to Chinese pig breeds with fast growth rate and high feed efficiency. Landrace originates from Denmark, and Yorkshire was bred through the cross between Asian breeds and British local breeds in past centuries [21]. Although Landrace and Yorkshire can be distinguished each other according to body size and ear morphology, they have similar selection direction in most economic traits. In this study, the principal component analysis (PCA) followed Paschou et al. [22] showed four breeds in this study are generally independent except that Songliao is genetically close to Landrace (S1 Fig.).

### SNP genotyping and data filtering

Genomic DNA samples from all of the pigs were extracted from ear tissue using a standard phenol/chloroform method. All of the DNA samples were analyzed by spectrophotometry and agarose gel electrophoresis. The genotyping platform used was Infinium II Multisample assay (Illumina, San Diego, CA). SNP arrays were scanned using iScan (Illumina, San Diego, CA) and analyzed using BeadStudio (Version 3.2.2, Illumina, San Diego, CA). The entire tissue sampling procedure was carried out in strict accordance with the protocol approved by the Animal Welfare Committee of China Agricultural University (Permit Number: DK996).

We implemented a quality control procedure to ensure the high data quality by (1) removing SNP loci with call rate less than 0.95 and unknown position, (2) removing SNP loci with minor allele frequency (MAF) less than 0.05 and (3) discarding the individuals with call rate less than 0.90. Following quality control, we imputed the missing genotypes and inferred haplotypes for the haplotype-based methods (LRH and XPEHH) using BEAGLE [23]. In addition, HAPLOVIEW v4.1 [24] was used to estimate linkage disequilibrium (LD) in four breeds, respectively.

### Methods for Detection of selection signature

Four methods, LRH, Tajima’s D，XPEHH and F_ST_, were implemented to detect the selection signatures. Tajima’s D and F_ST_ directly handle the SNP genotype, while XPEHH and LRH mainly use phased data. Among them, XPEHH and F_ST_ need to first define observed and reference population. In this study, Landrace was selected as the common reference population as it was involved in the cultivation of Songliao. Accordingly, three breed pairs of Yorkshire-Landrace (Y-L), Songliao-Landrace (S-L) and Rongchang-Landrace (R-L) were used for further analysis. For each breed pair, the common SNPs for one breed pair were unified before implementing XPEHH and F_ST_.

**LRH and XPEHH Analyses.** Both LRH and XPEHH are based on the ‘Extended Haplotype Homozygosity’ (EHH) algorithm to detect selection signatures. For LRH, the program Sweep v.1.1 was implemented to compute the ‘Relative Extended Haplotype Homozygosity’ (REHH) statistic and the core regions were defined as the chromosome fragments including at least 3 SNPs [25]. For XPEHH, the program at http://hgdp.uchicago.edu (coded by Joe Pickrell) was employed. The negative XPEHH scores suggest that selection occurred in the reference population, whereas the positive scores suggest the same about observed population. As the genetic distance between adjacent SNPs is needed for the calculation of XPEHH, a chromosome segment of 1Mb was straightly converted as 1 centiMorgan (cM).

**Tajima’s D and F**_**ST**_ **Analyses.** As a typical method for detecting selection signature [18], Tajima’s D considers the difference between the mean pairwise difference and the number of segregating sites in nucleotide polymorphism data. The test statistic equals zero for neutral variation, is positive when an excess of rare polymorphism is caused by recent balancing selection for multiple alleles and is negative when the excess of high-frequency variants suggests selective sweep. To reveal the divergent selection in this study, a two-step process of F_ST_ proposed by Gianola et al. [17,26] was employed to identify selection signatures based on population differentiation. The F_ST_ value ranges from 0 (identical population) to 1 (complete differentiation).

### Identifying potential selection signatures

As a widely used test of neutrality, Tajima’s D has the appealing property that its empirical distribution approximately follows a standard normal distribution after normalization [27]. Similarly, in this study, the empirical distribution of F_ST_ also approximately follows a standard normal distribution after the normalization of the square root of F_ST_ values [26]. For the LRH test, the across genome REHH values were ordered into 20 bins according to their frequency. After normalizing each bin by log-transformation, the LRH scores approximately follows a standard normal distribution [25]. In likewise, XPEHH approximately follows a standard normal distribution after normalization as well [19]. Hence, the significance test based on normal distribution was performed in these four methods to hunt the potential selection signatures in this study. Considering the multiple testing, False Discovery Rate (FDR) was implemented to control the false positive rate [28–30]. The test statistic values with FDR less than 0.1 for each method were outlier signals in this study, and extending 250 kb towards the upstream and downstream directions of one outlier would be a potential selection region (PSR).

### Gene annotation

To further control the false positive rates of the detection of selection signatures, we include the potential selection region as a candidate if FDR in one employed method is below 0.05 or if FDR in two or more employed methods is below 0.1. Bioinformatics analyses were then carried out to reveal the potential biological function of genes harbored in candidate selection regions through the NCBI database (http://www.ncbi.nlm.nih.gov/gene/). To further explore the biological function of those candidate selection regions, the QTLs enrolled in Pig QTLdb (www.animalgenome.org) were gathered and compared with those candidate selection regions based on the putative location of the QTLs.

## Results

### Information of chip data

Following quality control and principal component analysis (S1 Fig.), 83, 72, 86 and 97 individuals and 47114, 28997, 45945 and 47569 SNPs corresponding to Landrace, Rongchang, Songliao and Yorkshire were retained for this analysis (Table 1). The genome heterozygosity for Landrace, Rongchang, Songliao and Yorkshire is 0.345, 0.320, 0.347 and 0.335 on average, respectively. There is no significant difference in heterozygosity between Chinese local breeds and Western breeds. As a typical representative of Chinese indigenous pig breed, Rongchang has the minimum number of qualified SNPs, which is in agreement with the investigation by Ai et al. (2013) [6], in which most of Chinese indigenous pig breeds have lower number of SNPs. Further linkage disequilibrium analysis indicates that average r^2^ in Rongchang (0.150) is lower than it in Landrace (0.251), Songliao (0.261) and Yorkshire (0.248). This might attribute that Illumina Porcine60KSNP chip was designed mainly according to the genomic information of European pig breeds. Correspondingly, the number of unified SNPs for the Rongchang-Landrace pair was lowest as well. As shown in Table 2, for the F_ST_ and XPEHH analyses, a total of 43890, 45500 and 27100 common SNPs, were used for the Songliao-Landrace (S-L), Yorkshire-Landrace (Y-L) and Rongchang-Landrace (R-L) breed pairs, respectively. The average distance between adjacent SNPs for Y-L and S-L was approximately 50 kb, while 79 kb for R-L.

**Table 1:** Summary of selection signatures detected by LRH and Tajima’s D in four pig breeds.

	Breed	Landrace	Rongchang	Songliao	Yorkshire
	No. SNP	47114	28997	45945	47569
LRH	Outlier (FDR<0.1)	409	263	405	412
	Potential selection region (PSR)	248	198	276	258
	The total length of PSR (Mb)	188.85	147.16	204.59	192.25
	Mean of CHF^1^ in genome/PSR	0.24/0.26	0.22/0.27	0.23/0.27	0.24/0.23
	SD of CHF in genome/PSR	0.19/0.19	0.18/0.18	0.18/0.19	0.20/0.19
Tajima’s D	Outlier /PSR (FDR<0.1)	114	103	128	114
	The total length of PSR (Mb)	57.00	51.00	64.00	57.00
	Mean of DAF^1^ in genome/PSR	0.49 /0.47	0.50 /0.53	0.49 /0.48	0.51 /0.44
	SD of DAF in genome/PSR	0.25 /0.52	0.23 /0.45	0.25 /0.60	0.24 /0.46

^1^ CHF: Core haplotype frequency, DAF: the absolute allele frequency difference.

**Table 2:** Summary of selection signatures detected by XPEHH and FST in three breed pairs.

	Breed pair^1^	Y-L	S-L	R-L
	Common SNPs	45500	43890	27100
XPEHH	Outlier^2^ (FDR<0.1)	240(139)	152(160)	157(91)
	PSR in observed population	28	30	57
	The total length of PSR (Mb)	24.97	21.84	38.67
	Mean of DAFP^3^ in genome/ PSR	0.134/0.200	0.153/0.215	0.203/0.343
	SD of DAFP in genome/ PSR	0.155/0.197	0.156/0.208	0.233/0.329
F_ST_	Outliers (PSR)^2^ (FDR<0.1)	77	88	63
	The total length of PSR (Mb)	38.50	44.00	31.50
	Mean of DAFP in genome/PSR	0.195/0.444	0.205/0.460	0.335/0.733
	SD of DAFP in genome/PSR	0.125/0.186	0.130/0.190	0.200/0.180

^1^Y-L, S-L and R-L represent breed pair of Yorkshire-Landrace, Songliao-Landrace and Rongchang-Landrace, respectively.

^2^Outlier detected in observed population of Yorkshire, Songliao and Rongchang, those for Landrace were in brackets.

^3^DAFP: the absolute allele frequency difference between observed and reference population for an assigned allele (allele_1).

### Empirical distribution of four test statistics

**Figure 1:**
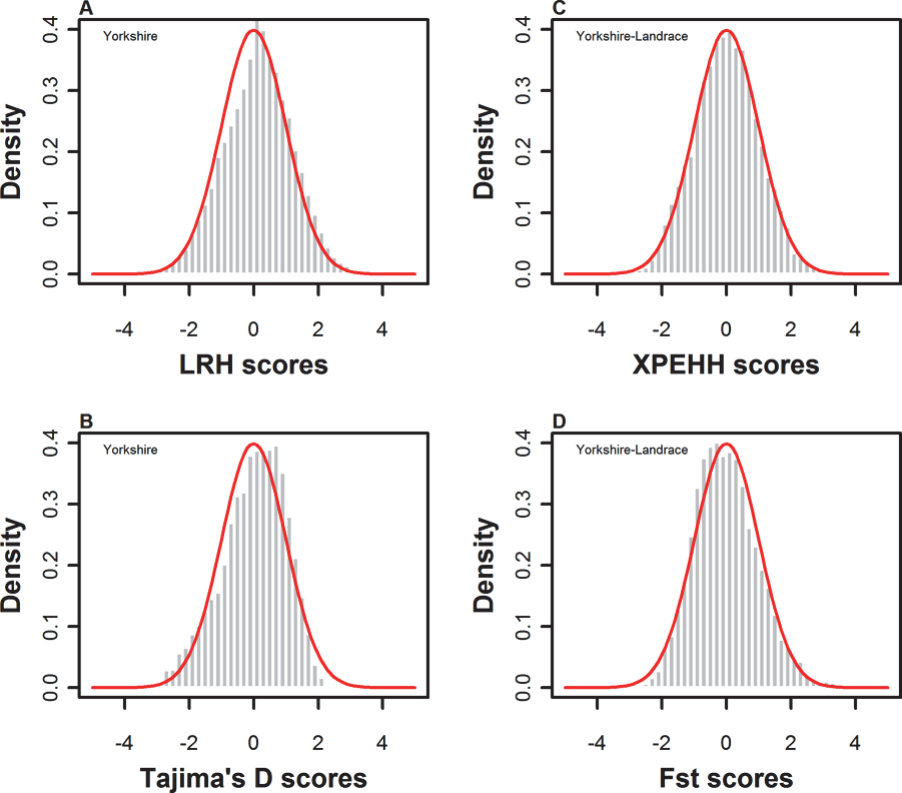
Empirical distribution of four test statistics in Yorkshire and breed pair of Yorkshire-Landrace.

The empirical distributions of all test statistics for each breed/breed-pair are clearly illustrated. Fig. 1 (A, B, C, D) plots the distributions of the LRH, Tajima’s D, XPEHH and F_ST_ statistic values across all autosomes for Yorkshire and Yorkshire-Landrace. The standardized LRH and XPEHH approximately followed a standard normal distribution, as pointed out by Sabeti et al. (2002, 2007) [19,25]. Similarly, the test statistics of Tajima’s D and F_ST_ after normalization roughly followed a standard normal distribution with a small skew. In addition, the distributions of the four test statistics indicated similar tendencies in other breeds/breed-pairs (S2 Fig.).

### Identification of selection signatures

**Figure 2:**
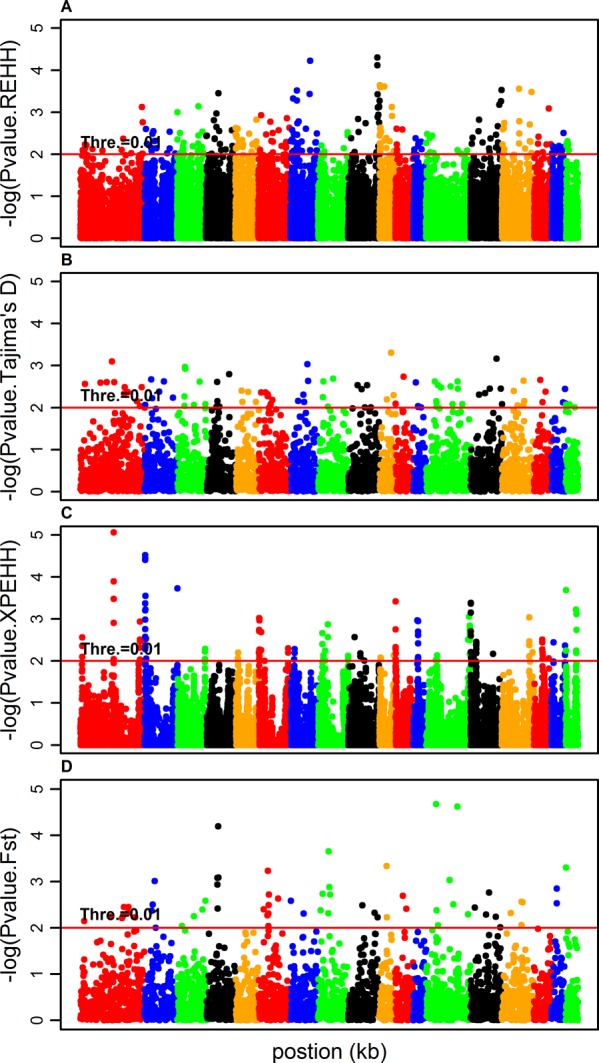
Genome-wide distribution of selection signatures detected by LRH, Tajima’s D, XPEHH and F_ST_ cross all autosomes in Rongchang.

**Figure 3:**
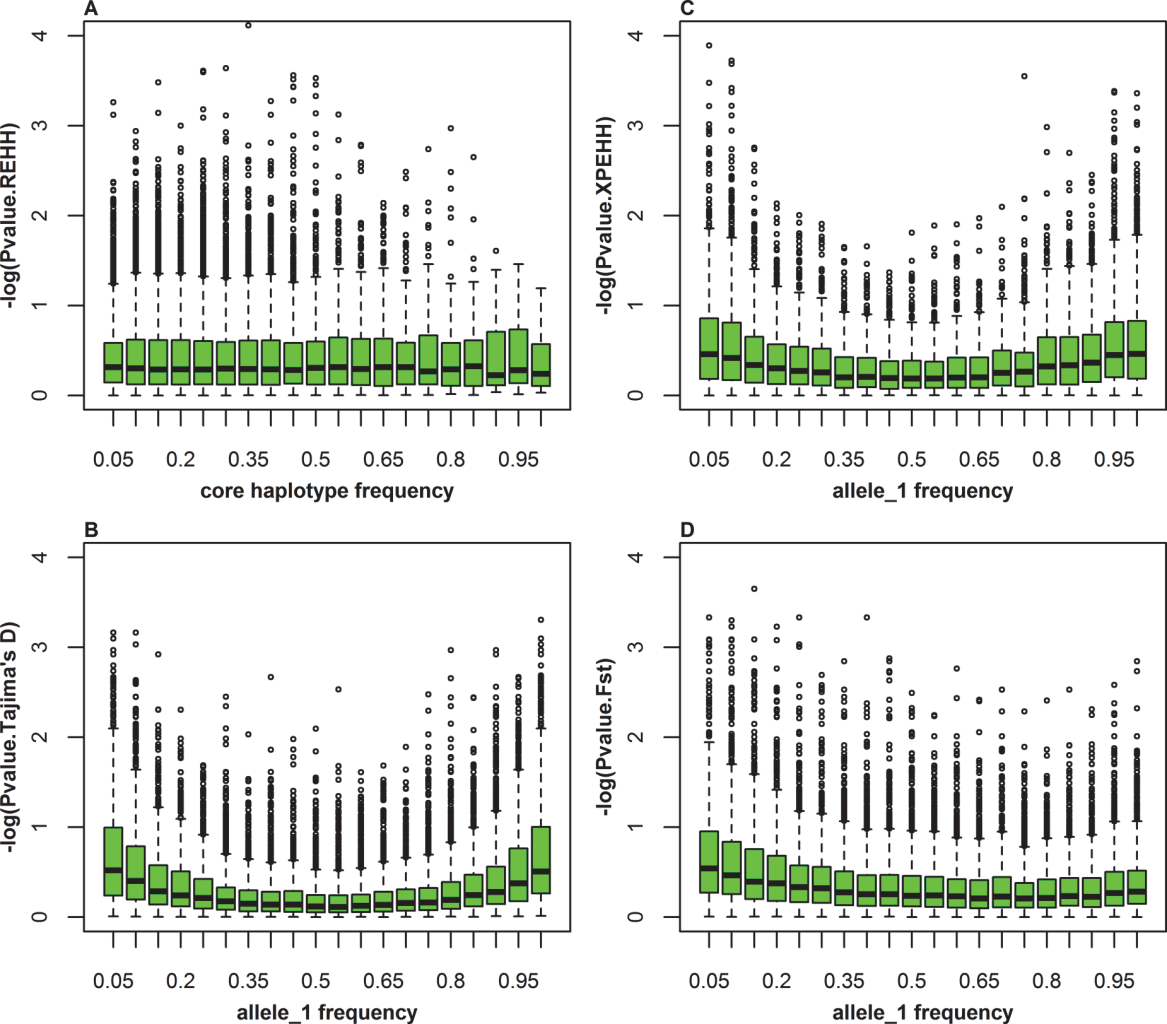
Distribution of –log(P-values) vs. haplotype/allele_1 frequency bins of 5% difference in Rongchang. Allele_1 is one assigned allele in each locus.

Fig. 2 (S3–S5 Figs.) depicts the genome-wide distribution of the outliers on each autosome that were detected separately by four approaches in Rongchang, Landrace, Songliao and Yorkshire. We divided the assigned allele/core haplotypes into a series of 0.05 bins according to their frequencies and used box-and-whisker plots to depict the distribution of the outliers (Fig. 3, S6–S8 Figs.). For LRH, it is obviously from Fig. 3A that the outliers were concentrated in the bins with low to moderate haplotype frequencies. This proved that LRH is effective for the detection of haplotypes under ongoing or incomplete selective sweeps, as reported by Sabeti et al. (2002) [25]. In contrast, other outliers detected by Tajima’s D, XPEHH and F_ST_ were concentrated on loci with low or high frequencies in all breeds (Fig. 3, S6–S8 Figs.), indicating that these methods are efficient for the identification of selection signatures under the complete selective sweeps.

Considering the phenomenon that core haplotypes harboring beneficial alleles undergoing selection usually have a high frequency, as noted by Sabeti et al.(2002) [25], we finally identified that the core haplotypes with frequencies greater than 0.25 and their corresponding REHH value with FDR below 0.1 were outliers under recent positive selection. Table 1 further summaries the outliers detected by LRH and Tajima’s D, respectively. As a haplotype-based method, LRH identified a total of 409, 263,405 and 412 core haplotypes as outliers in the four breeds, Landrace, Rongchang, Songliao and Yorkshire, respectively. These outliers correspond to 248, 198, 276 and 258 potential selection regions, spanning the length of 188.85 Mb, 147.16 Mb, 204.59 Mb and 192.25 Mb in Landrace, Rongchang, Songliao and Yorkshire, respectively. For Tajima’s D, each autosome was divided into a series of non-overlapping, consecutive, 500 kb windows. In total, 114, 103,128 and 114 outliers with 57.00 Mb, 51.00 Mb, 64.00 Mb and 57.00 Mb potential selection regions were identified in Landrace, Rongchang, Songliao and Yorkshire, respectively (Table 1). Although the mean of the absolute allele frequency difference at the locus at the genome level was very close to that in outlier for Tajima’s D, the standard deviation of the absolute allele frequency difference was double in outlier, which resulted in a coefficient of variation in outlier windows two times higher than the one in the genome (Table 1). This reflected that the alleles under selection usually presented a high frequency variation towards beneficial mutation fixation compared with the other alleles in genome.

Table 2 shows the selection signatures detected by XPEHH and F_ST_ in the three breed pairs of Y-L, S-L, and R-L when Landrace was treated as a common reference population. For XPEHH analysis, 379, 412 and 248 outliers were detected in three breed pairs, Y–L, S-L and R-L, respectively. Taking Y-L as an example, 240 positive XPEHH values out of 379 outliers indicated that selection occurred in observed population (Yorkshire), and the other 139 outliers with negative XPEHH values suggested selection in the reference population (Landrace). For further analysis, the outliers detected in each breed pair were assigned to each breed, and the potential selection region was defined as a region of 500kb around an outlier identified by XPEHH. After merging regions of overlap, 28, 30 and 57 potential selection regions were identified in three observed breeds, Yorkshire, Songliao and Rongchang, respectively. For the Landrace selection signatures, a total of 139 outliers fell into 30 potential selection regions when Yorkshire was treated as the reference population. For F_ST_, a total of 77, 88 and 63 potential selection regions were detected from the three breed pairs, Y-L, S-L and R-L, respectively. Table 2 also shows that both the mean and standard deviation of the absolute frequency difference on one assigned allele (allele_1 in Table 2 and Fig. 3) in two of the populations in outlier regions/windows were higher than those in all regions/windows for XPEHH and F_ST_. Particularly, the breed pair of Rongchang-Landrace (R-L) indicated larger difference compared with other breed pairs, implying different selection direction in Rongchang and Landrace.

### Candidate selection regions

To correctly reflect the footprints of selection, the potential selection region with FDR less than 0.05 for one method or FDR less than 0.1 for two or more methods were defined as a candidate selection region in this study. Finally, a total of 159, 127, 179 and 159 candidate selection regions with average lengths of 0.80 Mb, 0.73 Mb, 0.78 Mb and 0.73 Mb were identified in Landrace, Rongchang, Songliao and Yorkshire, respectively (Table 3, S9 Fig.). Overall, approximately 128.00 Mb, 92.38 Mb, 130.30 Mb and 115.40 Mb selection regions were detected in the four pig breeds, accounting for approximately 3.74–5.33 percent of the genome across all autosomes. Note that Rongchang, a representative Chinese indigenous pig breed, shared approximately 10.63 Mb candidate selection regions with the representative Chinese cultivated breed, Songliao. This is longer than the overlapping regions between Rongchang and Yorkshire. In addition, there are approximately 9.20 Mb candidate selection regions shared by Songliao and Yorkshire, which is longer than the length shared by Yorkshire and Rongchang but shorter than that shared by Rongchang and Songliao.

**Table 3:** Summary of whole genome candidate selection regions (Mb).

	Landrace		Rongchang		Songliao		Yorkshire	
Chr.	Number of regions	Length (Average)	Number of regions	Length (Average)	Number of regions	Length (Average)	Number of regions	Length (Average)
1	17	10.42(0.61)	12	7.52(0.63)	11	7.98(0.73)	14	9.27(0.66)
2	8	4.43(0.55)	7	4.68(0.67)	8	5.14(0.64)	14	10.60(0.76)
3	8	7.93(0.88)	8	5.83(0.73)	10	6.30(0.63)	11	8.03(0.73)
4	14	8.69(0.62)	9	6.17(0.69)	10	7.98(0.80)	11	6.96(0.63)
5	9	7.70(0.86)	2	1.24(0.62)	11	6.71(0.61)	7	4.21(0.60)
6	12	10.82(0.90)	13	9.96(0.77)	10	7.69(0.77)	10	8.23(0.82)
7	5	5.57(1.11)	11	9.34(0.85)	9	9.77(1.08)	14	9.34(0.67)
8	10	9.70(0.97)	8	4.52(0.56)	11	6.92(0.63)	4	2.40(0.60)
9	10	7.82(0.78)	10	6.39(0.64)	14	9.60(0.69)	8	5.68(0.71)
10	7	6.60(0.94)	8	8.27(1.03)	9	6.22(0.69)	12	8.34(0.69)
11	5	4.58(0.92)	3	1.73(0.58)	7	4.70(0.67)	8	7.42(0.93)
12	4	3.38(0.84)	3	2.00(0.67)	4	2.13(0.53)	5	4.14(0.83)
13	12	9.54(0.80)	10	6.68(0.68)	17	11.96(0.70)	8	5.33(0.67)
14	15	15.74(1.05)	9	6.84(0.76)	14	10.38(0.74)	11	9.32(0.85)
15	10	6.55(0.66)	6	5.29(0.88)	14	10.36(0.74)	8	5.75(0.72)
16	6	4.70(0.78)	3	2.46(0.82)	7	7.38(1.05)	4	2.71(0.68)
17	4	3.02(0.76)	2	1.21(0.61)	7	4.67(0.67)	6	3.22(0.54)
18	3	2.81(0.94)	3	2.26(0.75)	6	4.39(0.73)	4	4.39(1.10)
Total	159	128.0(0.80)	127	92.38(0.73)	179	130.3(0.78)	159	115.4(0.73)

### Genomic annotation

Based on the findings of selection regions, the candidate genes and QTLs harbored in the selection regions were revealed. The results of enrichment analysis did not show any intuitive information on selection. We noted that 33, 24, 26 and 27 candidate selection regions corresponding to Landrace, Rongchang, Songliao and Yorkshire were mapped in the gene deserts, which accounted for approximately 30 percent of all candidate selection regions in four breeds, respectively (S1 Table). In addition, some genes identified in the candidate selection region are yet not annotated. Nonetheless, many genes and QTLs identified under selection were still observed in our list (S1 Table). Meanwhile, Table 4 and Table 5 shows that a series of genes and QTLs associated with economic and appearance traits were not only identified in candidate selection regions but also in potential selection regions.

**Table 4:** Some Candidate genes located in or overlap with potential/candidate selection regions in four pig breeds^1^.

Chr.	Position(bp)^2^	P-value. (method)^3^	Breed	Candidate Gene	Gene function[reference]
2	2774804–3416230	0.0037(LRH);	S	CPT1A	Regulate fatty acid metabolism in newborn pig[36]
	2416056–2916056	0.0094(XPEHH);	Y	CPT1A	
	2381862–3500000	<0.0001(XPEHH);0.0086(Tajima’s D);	R	CPT1A	
	32500001–33000000	0.0021(Tajima’s D);	R	FSHB	Related with reproduction
4	30597930–31097930	0.0064(F_ST_);	Y	TRHR	Related with average backfat thickness, daily gain, and carcass and meat quality traits[31]
	30597930–31097930	0.0064(F_ST_);	L	TRHR	
	55500001–56250161	0.0082(LRH);0.0017(Tajima’s D);	L	CA3	Related with intramuscular fat content and percentage of ham of pigs[40]
5	30597930–31097930	0.0072(F_ST_);	Y	LEMD3	Related with flat-eared trait
	30597930–31097930	0.0072(F_ST_);	L	LEMD3	
	49117898–49617898	0.0022(F_ST_);	R	PTHLH	As functional candidate genes for their effects on number and shape of teats in pigs[11]
6	0–500000	0.0104(Tajima’s D)	S	MC1R	Coat colour variation
	49500001–50302217	0.0046(F_ST_);0.0102(Tajima’s D);	R	FUT1	Immunity
7	35667316–36167316	0.0500(F_ST_);	L,Y	PPARD	Backfat thickness [35], Ear size[34]
	33853417–35000000	0.0018(LRH);0.0069 (Tajima’s D);	R	HMGA1	Growth and fatdeposition[41]
8	43500001–44000000	0.0106 (Tajima’s D);	Y	KIT	Responsible for the white color
	44500001–45000000	0.0104(Tajima’s D);	L,S	KIT	
	72248126–72748126	0.0090 (F_ST_);	Y	ADAMTS3	Body size[5]
12	22640196–25548982	0.0081 (XPEHH);	S	PPP1R1B	Related with brain development and neuronal functions
	22000000–23000000	0.0011 (XPEHH);0.0090 (Tajima’s D);	R	PPP1R1B	
15	142004615–143483521	0.0005(XPEHH);0.0076(LRH);	Y	IRS1	A component of the highly conserved IGF1signalling cascade pathway that regulates skeletal muscle growth in mammals.
	142004615–143483521	0.0005(XPEHH);0.0076(LRH);	Y	COL4A4	Bone and cartilage development
16	20716185–21261283	0.0038(LRH);	R	SLC45A2	Coat colour variation
	21304135–21804135	0.0016(F_ST_);	R	PRLR	Related with litter size; related with ejaculate volume, sperm concentration, percentage of live sperm, and number of live sperm in the ejaculate; related with lateral-lying-to-other-posture trait and percentage ofsow-terminated nursing trait[43]

^1^ All related genes located in or overlapped with candidate selection regions see S1 Table

^2^ This column presents the position of selection regions, the bold and italics represent the potential selection region close to the corresponding gene.

^3^ The significance level for genome-wide P-values of each method was based on the standard False Discovery Rate (FDR).

**Table 5:** QTLs overlapped with the candidate/potential selection regions 1.

Chr	Position(bp)	P-value (method)	Breed	QTL Name
1	7552695–8292112	0.0045(LRH);	S	Name = Body weight (birth);Name = Average daily gain (10 weeks-slaughter);
2	2774804–3416230	0.0037(LRH);	S	Name = backfat (13 weeks of age);Name = Percentage of backfat and leaf fat in carcass;
	1966738–3171459	0.0094(XPEHH);	Y	Name = backfat (13 weeks of age);Name = Percentage of backfat and leaf fat in carcass;
	2381862–3500000	<0.0001(XPEHH);0.0086 (Tajima’s D);	R	Name = backfat (13 weeks of age);Name = Percentage of backfat and leaf fat in carcass;
3	122483364–123500000	0.0082(XPEHH);0.0086(Tajima’s D);	Y	Name = Toll-like receptor 2 level; Name = pH 24 hr post mortem (ham);
4	30597930–31097930	0.0064(F_ST_);	Y	Name = Backfat (average) thickness—ultrasound;Name = Loin eye area (22 weeks of age);Name = Meat color score;Name = Average Daily Gain (EBV);
	30597930–31097930	0.0064(F_ST_);	L	Name = Backfat (average) thickness—ultrasound;Name = Loin eye area (22 weeks of age);Name = Meat color score;Name = Average Daily Gain (EBV);
5	32514953–33014953	0.0072(F_ST_);	L	Name = Ear weight;Name = Ear area;
	32514953–33014953	0.0072(F_ST_);	Y	Name = Ear erectness;Name = Ear size;
6	0–500000	0.0104(Tajima’s D)	S	Name = subjective pork flavor in lean;Name = Litter weight, total;
	49500001–50302217	0.0032(F_ST_);0.0102(Tajima’s D);	R	Name = Small intestinal Escherichia coli F18receptor;Name = CD4-positive leukocyte percentage;
7	38400663–39454060	0.0071(F_ST_);0.0092(Tajima’s D);	Y	Name = Backfat at last rib (26 weeks);Name = Ear size;Name = Ear erectness;
	33368223–35000000	0.0018(LRH);0.0070(Tajima’s D);	R	Name = Ear weight;Name = Average backfat thickness;
12	22000001–23000000	0.0011(XPEHH);0.0093(Tajima’s D);	R	Name = Phosphate level;Name = Cooling loss;Name = Total muscle fiber number
14	135865348–138460325	0.0021 (XPEHH);0.0007(LRH);0.0065(Tajima’s D);	L	Name = Meat color density;Name = Vaccenic acid percentage;
16	68000001–70110082	0.0017(LRH);	Y	Name = Arachidic acid percentage;Name = Total body fat tissue linear;

^1^Complete QTL list see S1 Table and the significance level for genome-wide P-values of each method was based on the standard False Discovery Rate (FDR).

**Genes overlap with candidate/potential selection regions**. With the available annotation of the pig genome, Table 4 summarizes a part of the candidate genes falling into or overlapping with the candidate/potential selection regions in this study. Among them, the 30.819–30.823 Mb selection region on SSC4 was detected by F_ST_ in Yorkshire and Landrace. This region harbors the thyrotropin-releasing hormone receptor (TRHR) gene, which plays an important role in regulating the hypothalamic-pituitary-thyroid axis and, as a G-protein-coupled receptor, is relevant with average backfat thickness, daily gain, and carcass and meat quality [31]. The 21.52–21.55 Mb selection region on SSC16 and 49.16–49.17 Mb selection region on SSC5 were only identified in Rongchang, and these two regions overlapped with the prolactin receptor (PRLR) gene and the parathyroid hormone-like hormone (PTHLH) gene, which were reported to be relevant with litter size and teat shape in pig [5,32]. Several genes related to pig coat color were also identified in our study, e.g. the solute carrier family 45 member 2 (SLC45A2) gene [5] located in the 20.71–20.75 Mb region of SSC16 was only found in Rongchang, while the KIT gene [4] located in the 43.55–43.59 Mb region of SSC8 was detected in Landrace, Songliao and Yorkshire, respectively. Another well-known coat color gene, MC1R [33], which could influence the synthesis of coat color in pigs, was found to overlap with the selection region in Songliao. In addition to genes influencing coat color suffering from selection, the genes related with ear morphology were also overlapped with our selection regions, e.g. the LEM domain containing 3 (LEMD3) gene associated with flat-eared morphology [5] was separately overlapped with the 32.50–33.50 Mb selection region in Yorkshire and 32.10–33.01 Mb in Landrace on SSC5. Additionally, the region of 35.67–36.17 Mb on SSC7, identified in Yorkshire and Landrace by F_ST_, overlapped with the peroxisome proliferator-activated receptor delta (PPARD) gene, which not only affects ear size but also plays an important role in backfat thickness [34,35].

**QTLs overlap with selection regions.** The Pig QTLdb database (http://www.animalgenome.org/cgi-bin/QTLdb/index) has collected almost all of the QTLs reported in the past decades and is now being updated. Table 5 summarized some of the QTLs located in or overlapped with the selection regions in our study, more details see S1 Table. Taking the 0–4.41 Mb selection region of SSC2 detected in Songliao, Yorkshire and Rongchang for instance, two QTLs influencing backfat thickness and leaf fat in the carcass and meat and carcass quality were mapped in this region. Simultaneously, this region also overlapped with the carnitine palmitoyltransferase 1A (CPT1A) gene, which plays an important role in the regulation of fatty acid metabolism in newborn pig [36], as shown in Table 4. Again for ear morphology, a series of QTLs related to ear erectness in Yorkshire, ear size in Landrace and ear weight in Rongchang were separately found to overlap with the 32.51–33.01 Mb selection regions in Landrace and Yorkshire on SSC5, and 38.40–39.45 Mb in Yorkshire and 33.36–35.00 Mb in Rongchang on SSC7. It should be noted that the 22.00–23.00 Mb selection region on SSC12 detected by XPEHH and Tajima’s D in Ronchang overlapped with the QTLs related to the total muscle fiber number. Similarly, QTLs related to meat color density overlapped with the 135.87–138.46 Mb selection regions on SSC14 in Landrace, which was also simultaneously detected by three methods, XPEHH, LRH and Tajima’s D, respectively.

## Discussion

Most studies of selection signatures have only implemented a single method, but different methods emphasize different information in the data and are sensitive to different categories of selection signatures [13,14]. Hence, only applying a single method to detect selection signatures might result in some unknown bias. In this study, we applied four methods, LRH, Tajima’s D, F_ST_ and XPEHH, to explore the selection signatures in Landrace, Rongchang, Songliao and Yorkshire pig breeds. In accordance with previous research [19], our results (Fig. 2) showed that the LRH test is effective for detecting ongoing selection signatures with low to moderate frequency, while the other three methods are efficient in revealing approximate or fixed selection signatures. The Tajima’s D test focuses on selection signatures where the change in allele frequency under selection occurred quickly in comparison with the unselected loci in the same population. This feature was evident in the large difference in allele frequency at SNPs in the outlier windows and the genome (see Table 1). XPEHH is sensitive to detect approximately completed selection signatures in which the selected haplotype/allele has approached or achieved fixation in one population but remains polymorphic in the other one [19].

In addition, we also noted that the low density SNPs and the short-range LD pattern in Ronchang may result in low efficiency of haplotype-based methods to detect selection signatures. Our results shows LRH detected much less outliers in Rongchang than in other three breeds, while those detected by Tajima’s D are very close (Table 1). Similarly, XPEHH detected less outlier in Rongchang-Landrace than in Yorkshire-Landrace, and close to those in Songliao-Landrace, which should have relatively less outliers considering the genetic connectedness of Songliao and Landrace. Comparing with Rongchang, Songliao is a recently cultivated pig breed, conceivably resulting in long-rang LD pattern. It makes Songliao was detected more outliers by Tajima’s D and F_ST_ in comparison with LRH and XPEHH (Table 1 and 2), likely bring higher false positive rates. It should be careful to use such methods in this situation.

According to the selection signature findings, our study exhibited the genetic diversity of Rongchang, Songliao, Landrace and Yorkshire, which was caused by geographic difference，introgression and demographic history [1,2,8]. Comparing with the overlapping selection regions between breeds, Landrace and Yorkshire shared the longest overlap of 11.52 Mb in selection regions as they both originated from Europe. As two well-known in commercial pig breeds, they have already experienced a relative long period of adaptive evolution to meet similarly commercial requirements. Rongchang is a typical Chinese indigenous breed that was domesticated in Sichuan basin in the southwest of China, and its white coat makes Rongchang different from most Chinese indigenous breeds that mainly have black coats. The isolated environment reduces the genetic connection with other breeds and maintains the unique Rongchang characteristics, which results in the fewer overlaps of selection signatures with the other western pig breeds. Songliao was cultivated through hybridization of the Duroc, Landrace and Chinese Minzhu (another famous Chinese indigenous breed with a black coat in northeast of China) in past decades. Therefore, the overlapping selection regions of Songliao with Landrace were longer than Landrace shared with Rongchang. Meanwhile, Rongchang shared longer selection regions with Songliao than with Landrace and Yorkshire due to the evolutionary trajectory of Chinese pig. This conclusion agrees with previous studies [37,38] and implies that wide diversity exist in various populations due to geographic difference and various selection preference.

Most of the important economic traits in pig have been suffering from strong selection pressure according to previous research [39], which is also supported by our findings. A series of genes relevant to growth, meat quality, fatty metabolism and fertility were found to be under selection in this study (S1 Table, Tables 4, 5). Among them, the CA3 gene that located in 56.19–56.20 Mb on SSC4 was relevant to the intramuscular fat content and percentage of ham of pigs in accordance with previous research [40]. Correspondingly, the candidate selection region of 55.50–56.25 Mb in Landrace was identified by LRH and Tajima’s D, respectively. Similarly, the HMGA1 gene [41] related with the fat deposition was found in the candidate selection region in Rongchang, which was also detected by LRH and Tajima’s D, respectively. Note that these two genes, the CA3 gene and the HMGA1 gene, separately correspond to the typical characteristics in Landrace and Rongchang, especially the fat deposition in Chinese local breed. Additionally, the genes harbored in potential selection regions were also relevant to the economic features of pig breeds. For example, three candidate genes (FSHB [42], PTHLH [11] and PRLR [43]) associated with reproduction traits were only identified in Rongchang, implying that the genetic mechanism for reproductive capacity in Rongchang may be different from others (Table 4). As an establised sweep, IGF2 should have been detected under selection in this research, but unfortunately, our results could not provide further support as reported in other studies [9] because the fine location of this gene is still not clear in the current pig genome.

**Figure 4:**
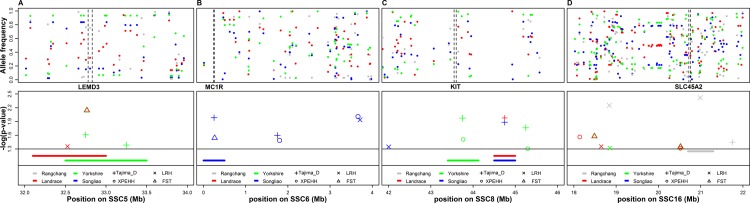
Patterns of genetic variation associated with LEMD3, MC1R, KIT and SLC45A2 genes. Variation in breed allele frequencies of SNPs at the potential selection region for ear morphology/coat color on SSC5, SSC6, SSC8 and SSC16 (color coded by the pig breeds to which they belong), Genomic distribution of potential selection region as measured by four methods, each symbol represents a candidate selection region identified by corresponding methods.

Ear morphology and body length are two important indicators that distinguish Landrace and Yorkshire. Accordingly, the 32.09–33.50 Mb candidate selection region on SSC5 in Yorkshire and Landrace harbored two candidate genes (WIF1 and LEMD3) that were reported to be relevant to bone development and ear morphology [44,45]. Fig. 4A demonstrates the selection regions that contains LEMD3 gene, the selection regions were identified by Tajima’s D, LRH and F_ST_ (P-value <0.05) in Landrace and Yorkshire, respectively. After the correction of multiple testing, the potential selection regions identified by F_ST_ is still significant and this method is properly sensitive in divergent selection in according to previous research [15]. This phenomenon not only demonstrates the efficiency of the strategy of multiple methods, but also suggests that the ear morphology has been suffered different selection pressure between Landrace and Yorkshire. Ren et al. (2011) investigated PPARD related with ear morphology and underwent a selective sweep signal in Erhualian, a famous Chinese indigenous breed with large and floppy ears, simultaneously, they detected PPARD gene associated with backfat thickness due to the pleiotropism [34]. However, in this study, we only detected PPARD gene under selection in Landrace and Yorkshire by F_ST_ without correction of multiple testing. In addition, the ADAM metallopeptidase with thrombospondin type 1 motif, 3 (ADAMTS3) gene [5], which is involved in body size, were found in the potential selection region in Yorkshire. Correspondingly, the ADAMTS12 gene [5] that was also relevant with body size was detected in Landrace with extreme statistical values when XPEHH and F_ST_ were separately performed. This may provide support for the difference in their body size.

Coat color is one of important features for pig breeds as well. KIT and MC1R that associated with coat color have been investigated to suffer selection in pig and chicken [4,46]. In this study, KIT gene overlaps with or closes to the potential selection region in Yorkshire, Landrace and Songliao through the detection of Tajima’s D (Fig. 4C). However, there is no potential selection region overlapping with KIT gene in Rongchang. As to MC1R, the mutation at amino acid 240 deduced Duroc’s distinctive red coat, implying that only pigs with Duroc ancestry will contain this mutation allele in the MC1R gene [33,46]. Coincidently, MC1R gene was only detected under selection in Songliao in our study possibly attributed to the introgression of Duroc in the cultivation of Songliao (Fig. 4B). Wilkinson et al. (2013) collected 14 pig breeds with 24–34 individuals per breed to detect selection signatures using PorcineSNP60 chip, while they did not find KIT and MC1R gene under selection in European pigs. They attributed it to the poor coverage of the PorcineSNP60 chip [5]. This may be one explanation for the phenomenon that the KIT and MC1R genes were not detected under selection in Rongchang, only 28997 SNPs available. Another reason could be that the genetic mechanisms of white color in Rongchang are different from that in Yorkshire and Landrace (Fig. 4). Correspondingly, SLC45A2 gene was detected under selection by LRH in Rongchang, while it was not identified in Yorkshire and Landrace. However, when FDR was not carried out, SLC45A2 gene was identifed suffering from selection in these three white coat color breeds by three methods of LRH, F_ST_ and XPEHH respectively (Fig. 4D), which is in accordance with Wilkinson et al. (2013) [5].

So far, several researches have been carried out to identify selection signatures in pig [4–9]. Ai et al. (2013) [6], Wilkinson et al. (2013) [5] and Yang et al. (2014) [7] also detected selection signatures using Porcine SNP60BeadChips. However, Ai et al. (2013) [6] and Yang et al. (2014) [7] only reveal a few potential selection signatures. On the contrary, Wilkinson et al. (2013) reveal selection signatures completely through widely between-population and within-population analysis [5]. Accordingly, a series of well-known candidate genes were found and most of them are reproductive in this study, such as WIF1 and LEMD3. In addition, we note that those researches only collected about 30 individuals in each breed, and the small sample size may make them inefficient to detect selection signatures, e.g. MC1R gene were not identified under selection by Wilkinson et al. (2013) [5], while identified in our study. As to the selection signatures relevant to economic traits, the low reproducibility across different researches is a common phenomenon in farm animal chip data analysis. This may be caused by the high marker distance of Porcine SNP60BeadChips. Rubin et al. (2010) [20] and Amaral et al. (2010) [8] employed the pool sequencing data to detect selection signatures in pig and they also found a series of interest candidate genes. However, the analysis of pool sequencing data only can make use of the information from allele frequency, which is one of elements in detecting selection signature. We know the long range haplotype is also an important element in detecting selection signatures. Despite this, Rubin et al. (2010) also highlighted a few established selection signatures, such as KIT gene, and displayed a series of reliable evidences to support the inferences [20]. With the development of sequencing technique, it becomes promising to detect selection signatures using sequencing data, especially the individual resequencing data, which may improve the accuracy of selection signature detection through improving the density of SNPs [4]. While the sample size and coverage of sequencing also need to be taken into consideration as point out by Cutler et al. (2010) [47].

## Supporting Information

S1 Fig Scatter plots of the population structure of 338 individuals via principal component analysis. (TIFF)

S2 Fig Empirical distribution of four test statistics in Songliao, Rongchang, Landrance and breed pairs of Songliao-Landrace and Rongchang-Landrace. (TIFF)

S3 Fig Genome-wide distribution of –log(P-values) vs. physical distance in Landrace (TIFF)

S4 Fig Genome-wide distribution of –log(P-values) vs. physical distance in Songliao. (TIFF)

S5 Fig Genome-wide distribution of –log(P-values) vs. physical distance in Yorkshire. (TIFF)

S6 Fig Distribution of—log(P-values) vs. haplotype/allele_1 frequency bins of 5% difference in Landrace. (TIFF)

S7 Fig Distribution of—log(P-values) vs. haplotype/allele_1 frequency bins of 5% difference in Songliao. (TIFF)

S8 Fig Distribution of—log(P-values) vs. haplotype/allele_1 frequency bins of 5% difference in Yorkshire. (TIFF)

S9 Fig Genome map of candidate selection regions in four pig Breeds. (TIFF)

S1 Table The complete list of genes and QTLs overlapping with candidate selection regions in four pig breeds. (XLSX)
