# Supplementary figures and images for: A Genome Scan for Selection Signatures in Pigs
Source: PLoS One. 2015 Mar 10;10(3):e0116850. doi: 10.1371/journal.pone.0116850 (PMC4355907; doi:10.1371/journal.pone.0116850)

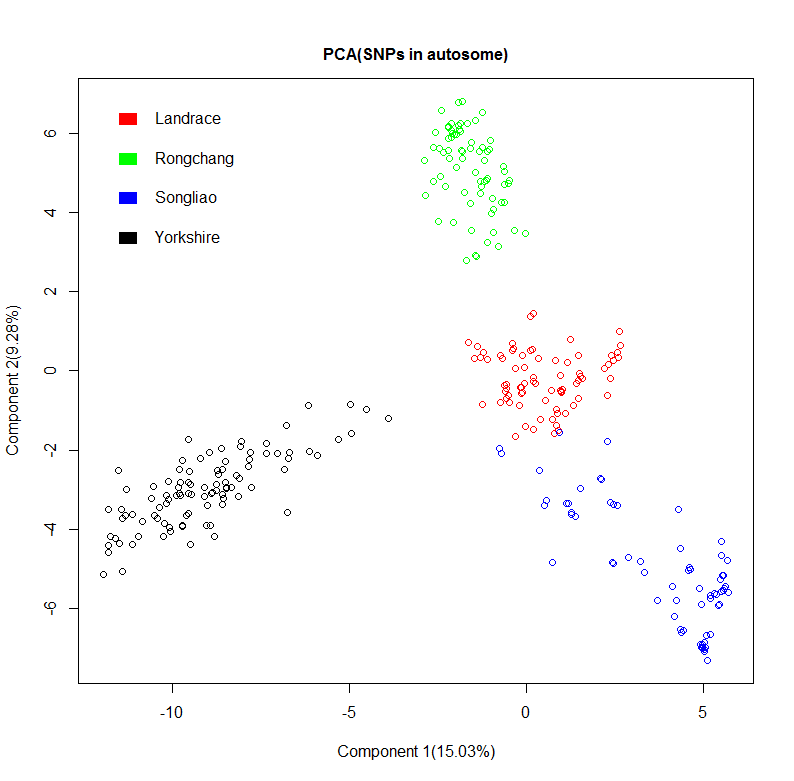

Supplement: S1 Fig — (TIFF) [file pone.0116850.s001.tiff]

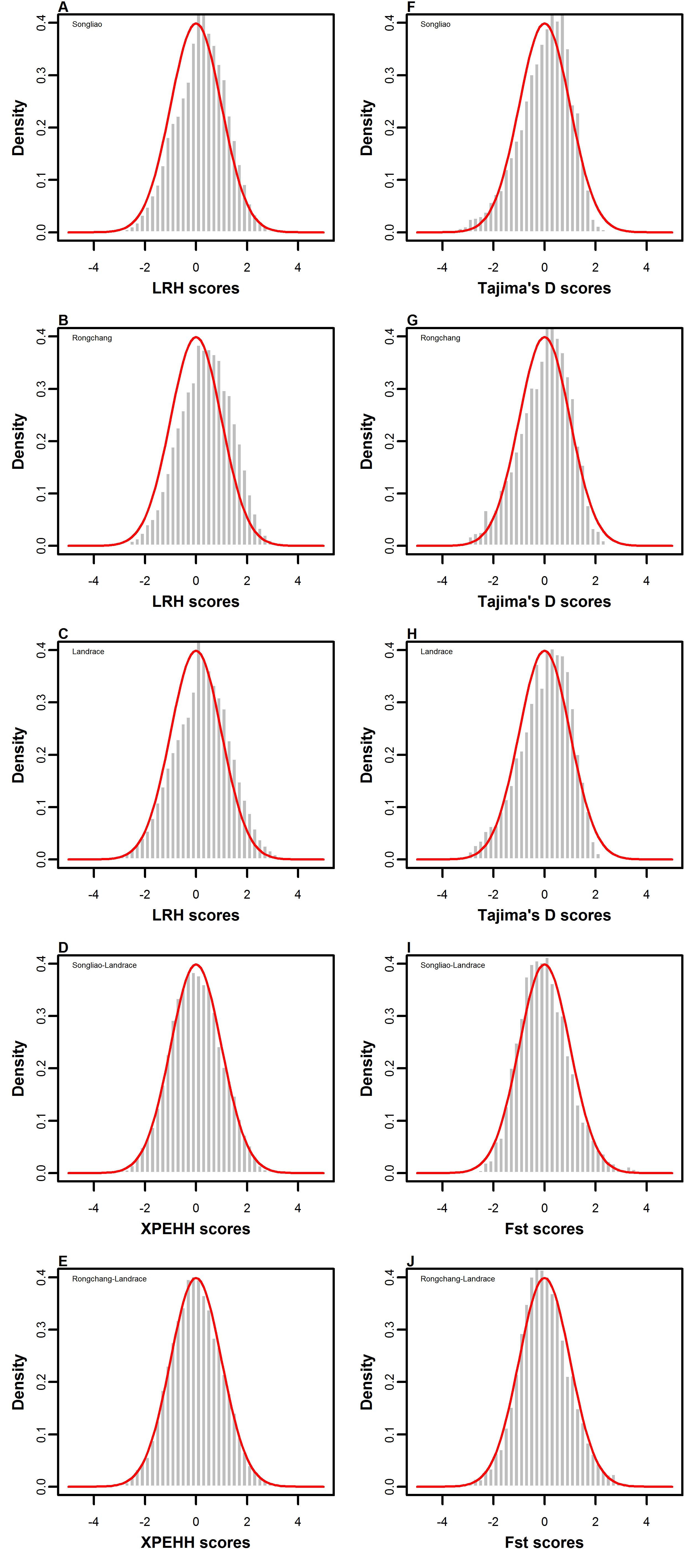

Supplement: S2 Fig — (TIFF) [file pone.0116850.s002.tiff]

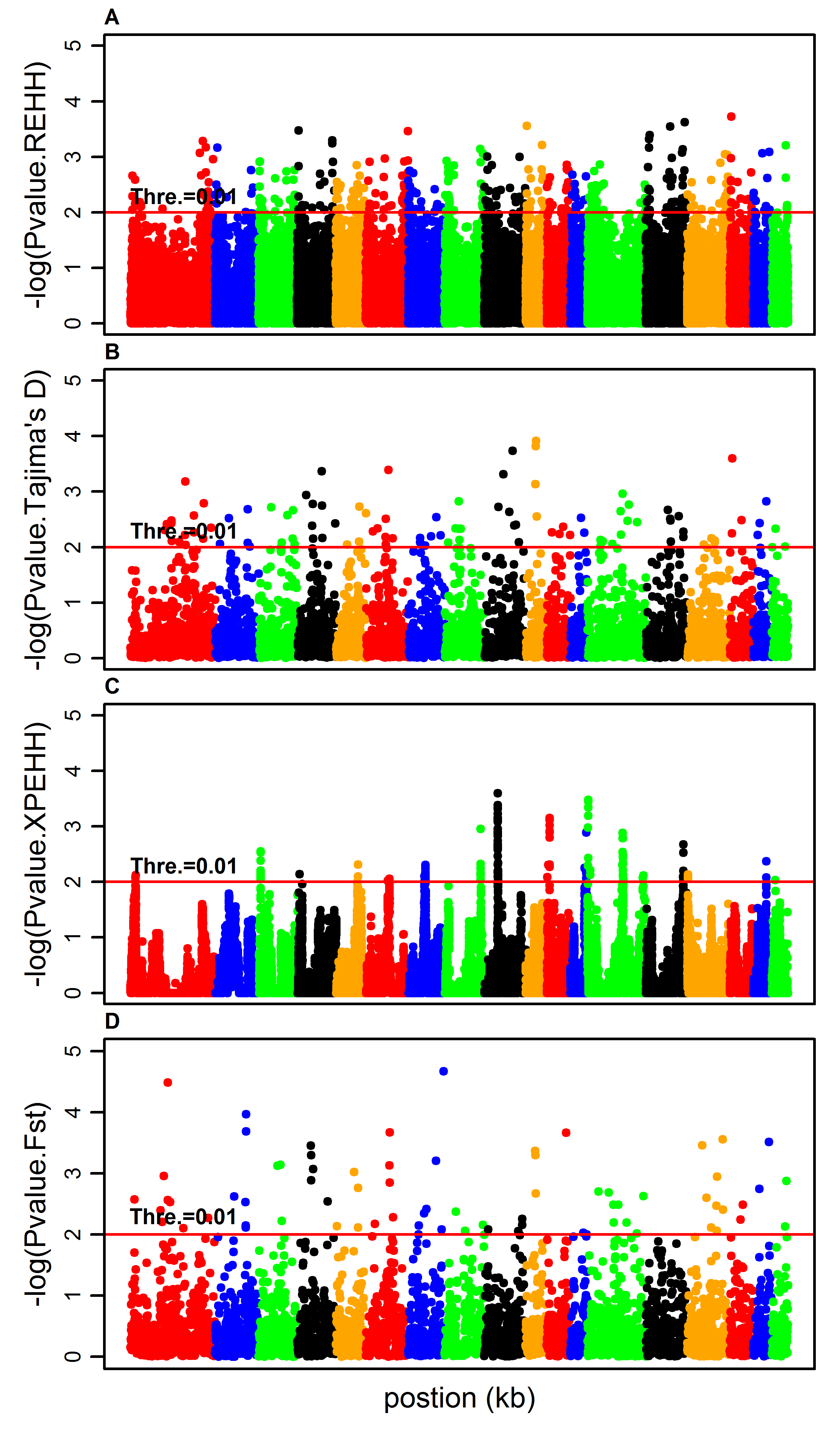

Supplement: S3 Fig — (TIFF) [file pone.0116850.s003.tiff]

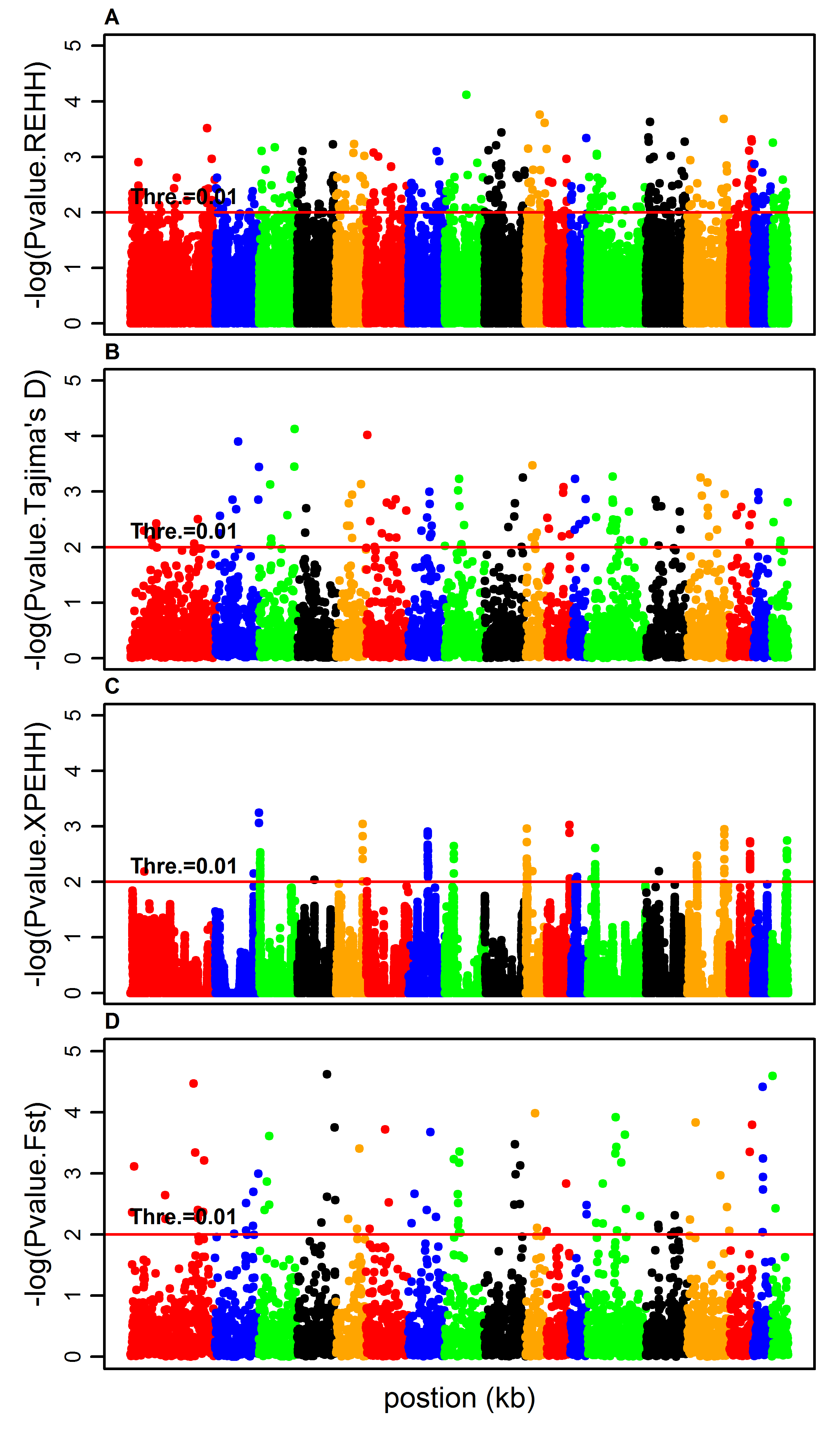

Supplement: S4 Fig — (TIFF) [file pone.0116850.s004.tiff]

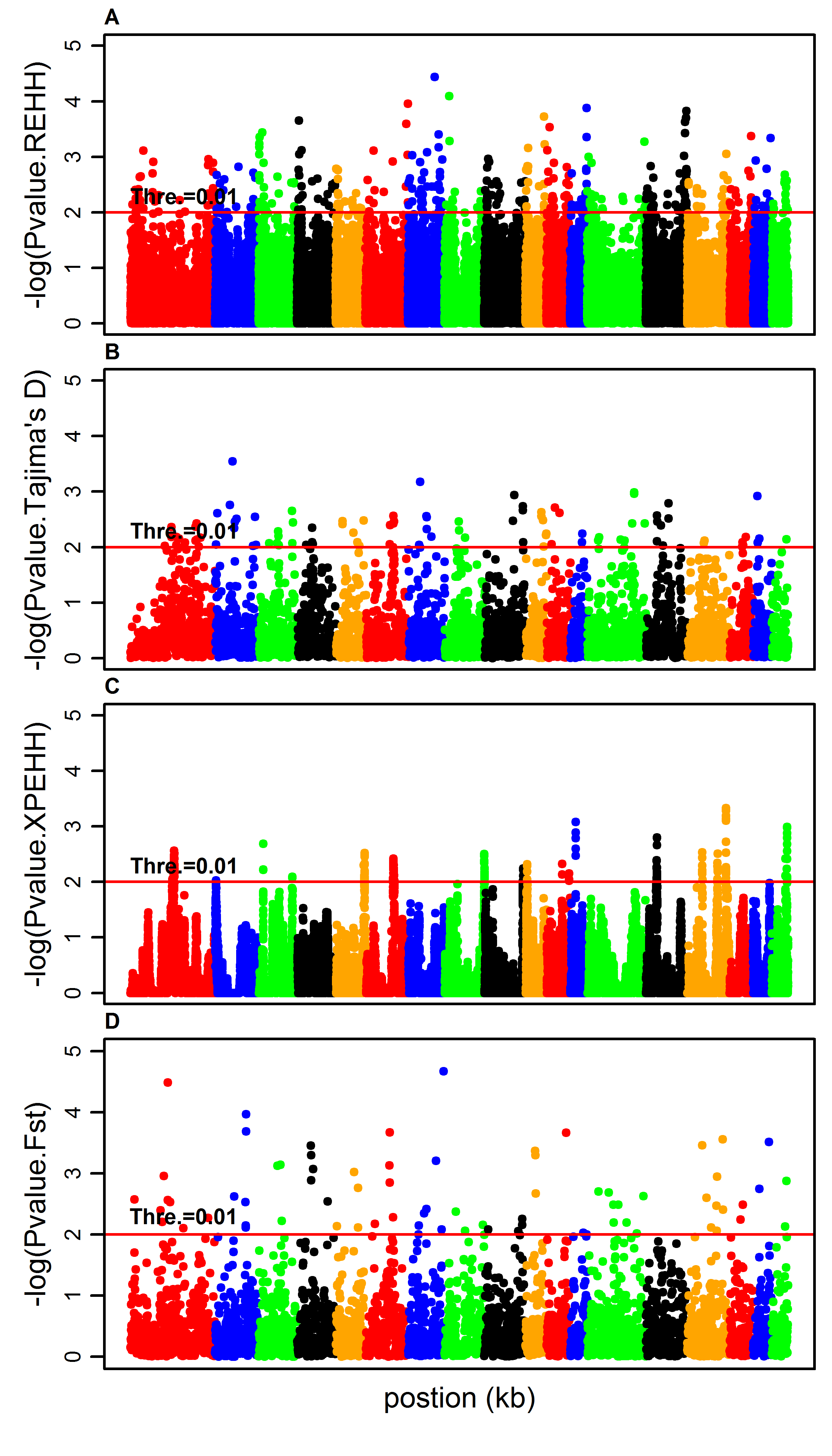

Supplement: S5 Fig — (TIFF) [file pone.0116850.s005.tiff]

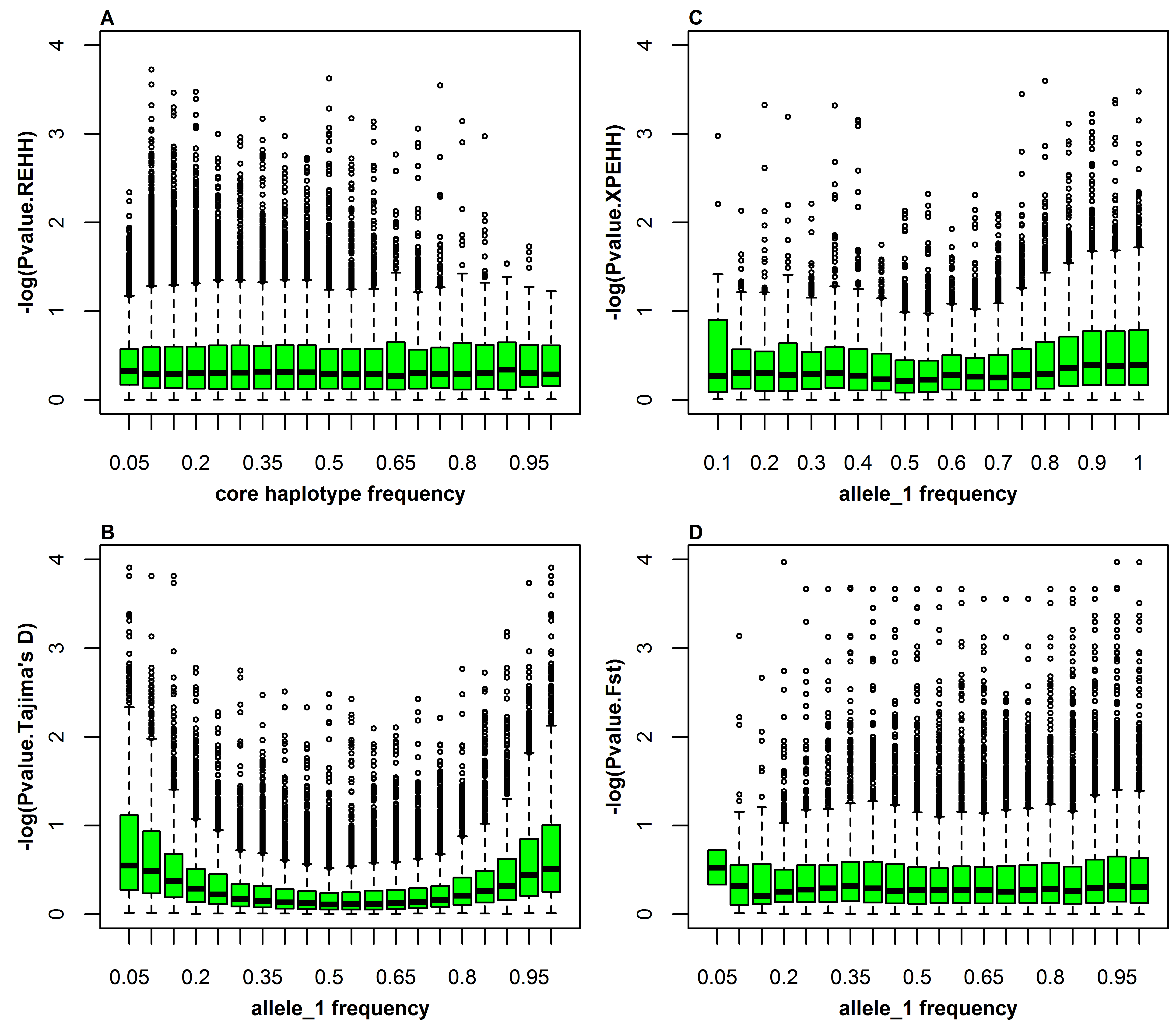

Supplement: S6 Fig — (TIFF) [file pone.0116850.s006.tiff]

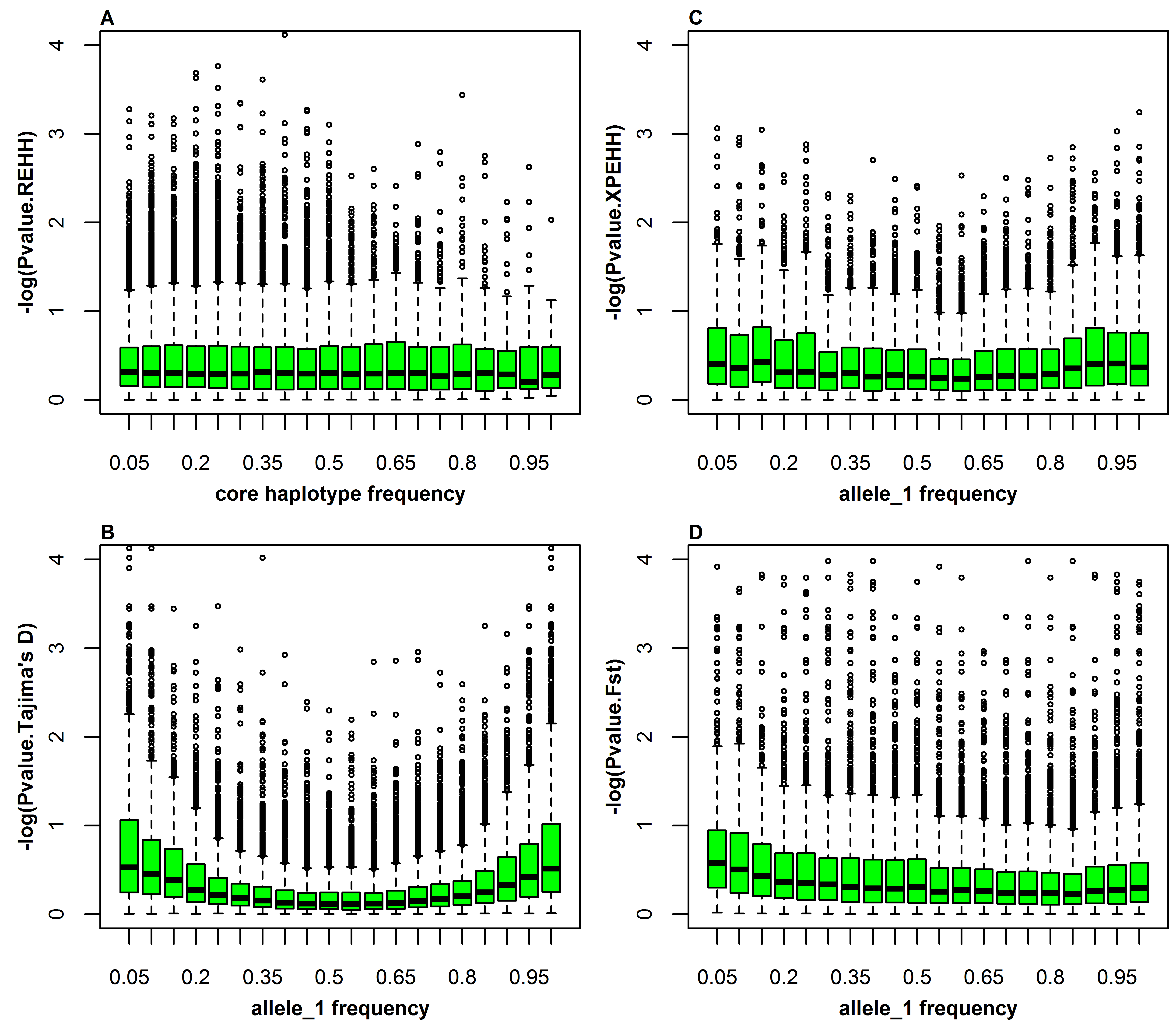

Supplement: S7 Fig — (TIFF) [file pone.0116850.s007.tiff]

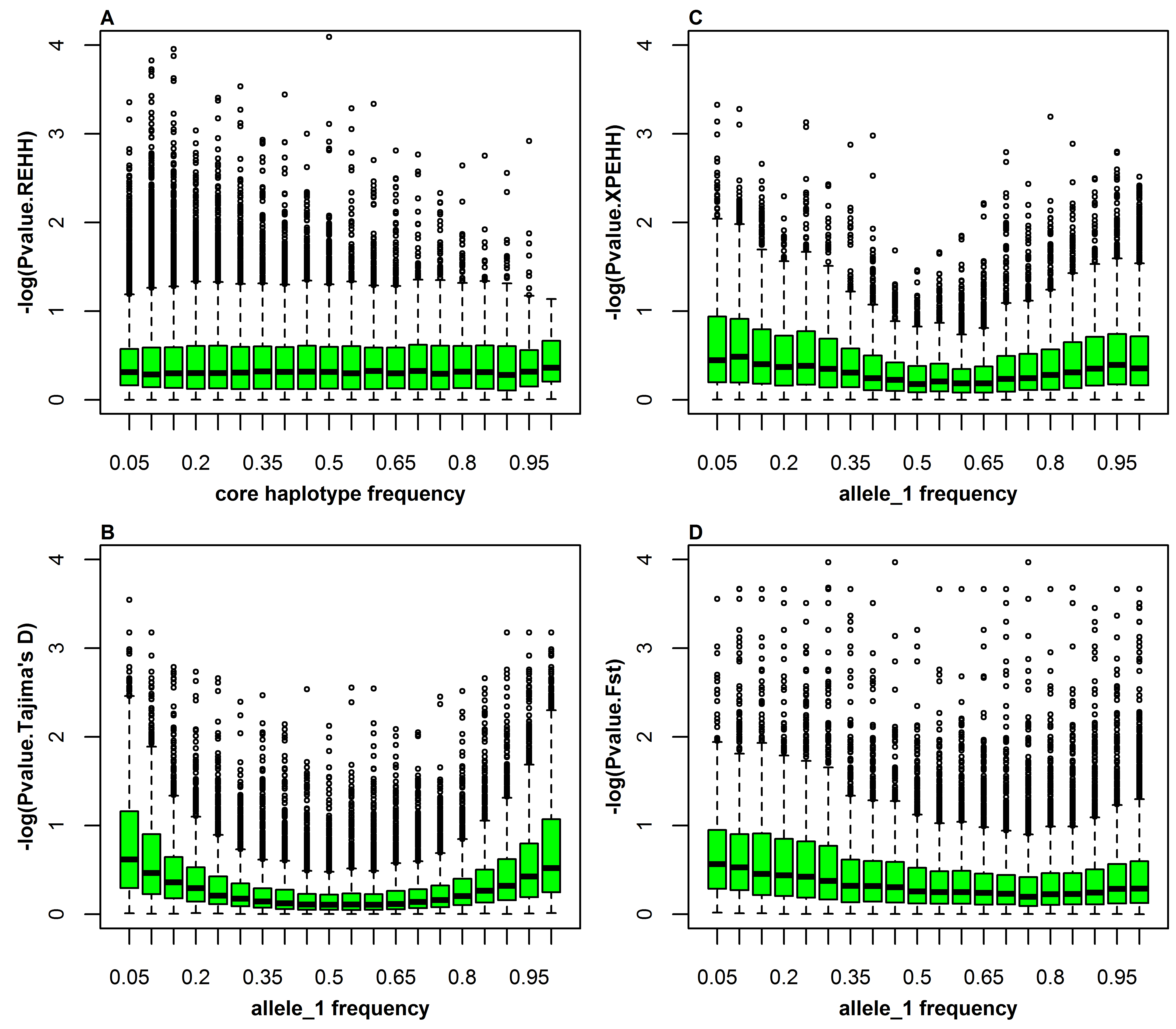

Supplement: S8 Fig — (TIFF) [file pone.0116850.s008.tiff]

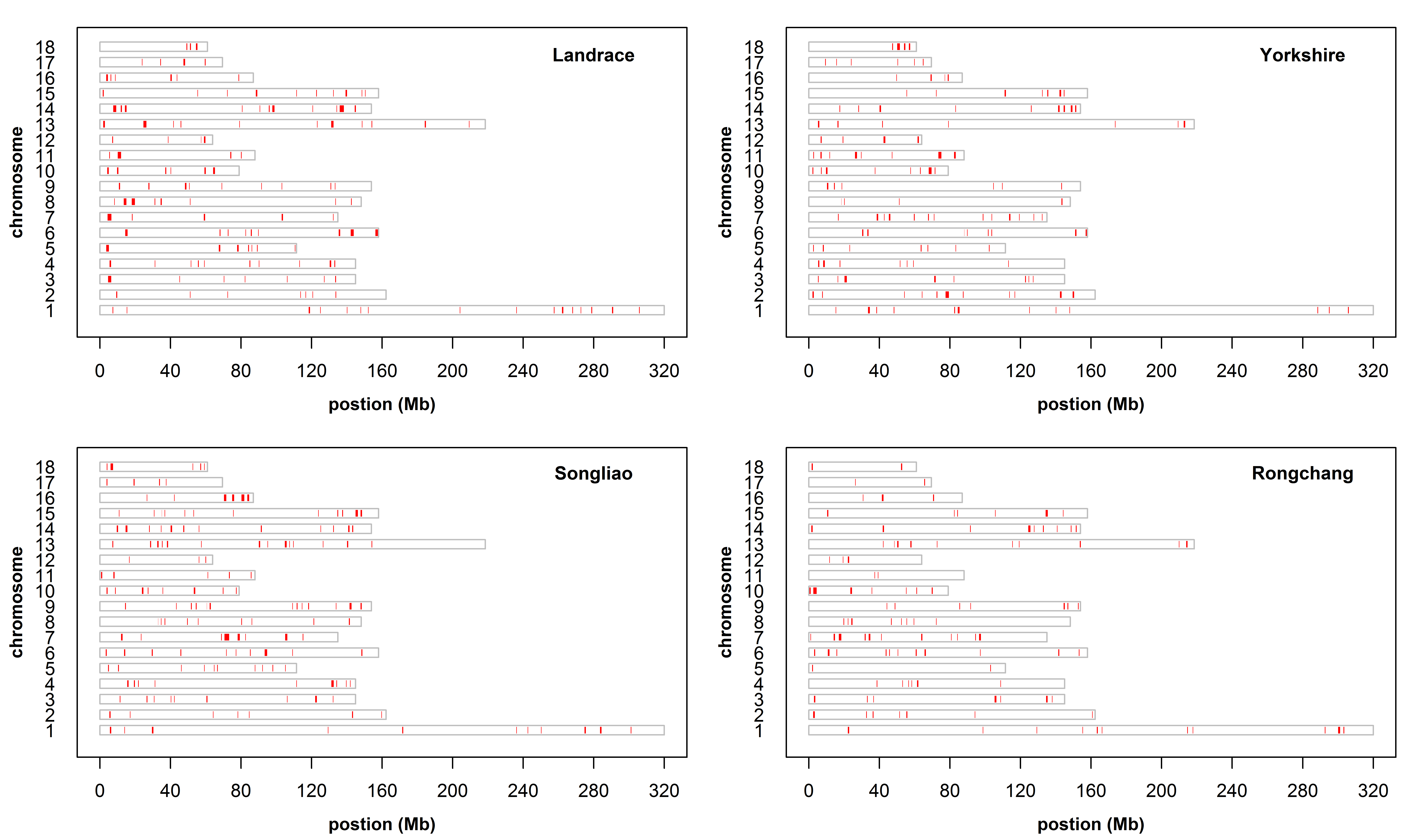

Supplement: S9 Fig — (TIFF) [file pone.0116850.s009.tiff]
